# MicroRNA modulation in complex regional pain syndrome

**DOI:** 10.1186/1479-5876-9-195

**Published:** 2011-11-10

**Authors:** Irina A Orlova, Guillermo M Alexander, Rehman A Qureshi, Ahmet Sacan, Alessandro Graziano, James E Barrett, Robert J Schwartzman, Seena K Ajit

**Affiliations:** 1 Pharmacology & Physiology, Drexel University College of Medicine, Philadelphia, PA 19102, USA; 2 Neurology, Drexel University College of Medicine, Philadelphia, PA 19102, USA; 3 School of Biomedical Engineering, Science, and Health Systems, Drexel University, Philadelphia, PA 19104, USA

**Keywords:** MicroRNA, biomarker, pain, CRPS

## Abstract

**Background:**

Aberrant expression of small noncoding RNAs called microRNAs (miRNAs) is a common feature of several human diseases. The objective of the study was to identify miRNA modulation in patients with complex regional pain syndrome (CRPS) a chronic pain condition resulting from dysfunction in the central and/or peripheral nervous systems. Due to a multitude of inciting pathologies, symptoms and treatment conditions, the CRPS patient population is very heterogeneous. Our goal was to identify differentially expressed miRNAs in blood and explore their utility in patient stratification.

**Methods:**

We profiled miRNAs in whole blood from 41 patients with CRPS and 20 controls using TaqMan low density array cards. Since neurogenic inflammation is known to play a significant role in CRPS we measured inflammatory markers including chemokines, cytokines, and their soluble receptors in blood from the same individuals. Correlation analyses were performed for miRNAs, inflammatory markers and other parameters including disease symptoms, medication, and comorbid conditions.

**Results:**

Three different groups emerged from miRNA profiling. One group was comprised of 60% of CRPS patients and contained no control subjects. miRNA profiles from the remaining patients were interspersed among control samples in the other two groups. We identified differential expression of 18 miRNAs in CRPS patients. Analysis of inflammatory markers showed that vascular endothelial growth factor (VEGF), interleukin1 receptor antagonist (IL1Ra) and monocyte chemotactic protein-1 (MCP1) were significantly elevated in CRPS patients. VEGF and IL1Ra showed significant correlation with the patients reported pain levels. Analysis of the patients who were clustered according to their miRNA profile revealed correlations that were not significant in the total patient population. Correlation analysis of miRNAs detected in blood with additional parameters identified miRNAs associated with comorbidities such as headache, thyroid disorder and use of narcotics and antiepileptic drugs.

**Conclusions:**

miRNA profiles can be useful in patient stratification and have utility as potential biomarkers for pain. Differentially expressed miRNAs can provide molecular insights into gene regulation and could lead to new therapeutic intervention strategies for CRPS.

## Background

Complex regional pain syndrome (CRPS) is a disabling chronic neuropathic pain syndrome that can affect one or more extremities. The broad spectrum of symptoms includes pain, inflammation, sensory dysfunction, impaired motor function, and trophic disturbances [1-4]. CRPS is subdivided into CRPS-I (reflex sympathetic dystrophy) and CRPS-II (causalgia), based on the absence or presence of documented nerve injury respectively [5]. The complex multifactor pathogenesis of CRPS includes inflammatory, vascular, sympathetic nervous system, cortical, and spinal mechanisms. The pathophysiology of CRPS is not completely understood and the diagnosis is based solely on clinical observations. Not all disease mechanisms are equally prominent in each patient and no single therapeutic modality is sufficient to attenuate all of the symptoms.

Small nonprotein coding endogenous ~22 nucleotide RNA molecules called microRNAs (miRNAs) have attracted considerable attention in an effort to dissect the molecular changes in various disease models. miRNAs play important roles in the regulation of gene expression and function by binding to the 3' untranslated region (3'-UTR) of target messenger RNAs (mRNAs) that, in turn, causes cleavage or repression of translation of these mRNAs. Each miRNA species regulates multiple genes, and most mRNA targets contain multiple miRNA binding sites within their 3'-UTR, suggestive of a complex regulatory network [6]. As aberrant miRNA expression is a common feature in a variety of human diseases, these molecules offer novel avenues for the identification of biomarkers and new opportunities for the discovery and validation of novel therapeutic targets [7].

It was recently demonstrated that miRNAs are present in the serum and plasma of humans and other mammals, such as rats, mice, cows and horses [8,9]. This finding opens up the feasibility of using miRNAs as biomarkers of disease. Though the stability of miRNAs in serum was the initial concern, it has now been demonstrated that these circulating miRNAs are protected from plasma RNase activity and are, in fact quite stable [8]. The existence of tumor-related miRNAs in serum indicates the potential usefulness of miRNAs as clinical diagnostic biomarkers of various cancers [10]. In another recent report, dozens of stable miRNAs were detected in saliva and two miRNAs were present in significantly lower levels in the saliva of patients with oral squamous cell carcinoma compared to control subjects [11]. Further evidence for the presence of miRNAs in body fluids came from an analysis of urine samples [12]. Four miRNAs were significantly elevated in urine from urothelial bladder cancer patients, demonstrating the utility of miRNAs as a noninvasive diagnostic option [13]. All of these studies illustrate the potential use of miRNAs as novel biomarkers amenable to clinical diagnosis in translational medicine [12,14-16]. Biomarkers can be used to determine the propensity to develop a disease, measure its progress, or predict prognosis [17]. In clinical trials, biomarkers can help in patient stratification and thereby increase the chances of a successful outcome by targeting the appropriate population. In addition, biomarkers can pave the way to individualize treatment and thereby usher in a new era in personalized medicine [18].

A number of studies have addressed miRNA changes in rodent models of inflammatory and neuropathic pain indicating an essential role for miRNAs in altering pain threshold [19-25]. Although deregulation of miRNA is now a well-established phenomenon in a number of human diseases including osteoarthritis pain [26], there are, to our knowledge, no published reports investigating the role of miRNA expression in patients with neuropathic pain. The objective of this study was to investigate differential expression of miRNAs in blood from patients with CRPS as an initial step to determine its utility as a novel approach to biomarker development. Correlation studies also were performed using miRNA profiles, inflammatory markers and other disease parameters. Eighteen miRNAs were significantly different between patients and control subjects compared to three inflammatory and immune related markers. Clustering of 60% of patients with CRPS on the basis of the miRNA profile suggests that clinically relevant stratification of the patient population is possible on the basis of alterations in miRNA expression.

## Methods

### Standard protocol approvals, registrations, and patient consents

All subjects were enrolled after giving informed consent as approved by the Drexel University College of Medicine Institutional Review Board.

### Inclusion and exclusion criteria

Patients with CRPS were recruited from the pain clinic of Drexel University College of Medicine and fulfilled the International Association for the Study of Pain (IASP) diagnostic criteria for CRPS [5]. Healthy control subjects were recruited from the general public. The exclusion criteria for all subjects included pregnancy, recent infection, lupus erythematosus, HIV/AIDS, rheumatoid arthritis, recent extracorporeal circulation (hemodialysis, bypass surgery, plasmapheresis), bone marrow transplant, immunosuppressive therapy, blood disorders (anemia, leukemia), thymectomy, or sarcoidosis.

### Patient evaluation

All patients with CRPS received a complete neurological examination and pain evaluation (performed by RJS). Overall pain levels were determined on a 0-10 numerical rating scale (NRS) (0 = no pain, 10 = worst possible pain). Additional information collected included duration of CRPS in years, age of onset, and medications (narcotics, antiepileptics, antidepressants, and anxiolytics). The presence of other conditions such as radiculopathy, heart disease, arthritis, irritable bowel syndrome, high blood pressure, seizure disorder, spinal disk disease, high cholesterol, generalized anxiety disorder, depression, gastroesophageal reflux disease, migraines, and thyroid disease was also noted. Medical history and self-reported values for height and weight were obtained from normal healthy control subjects. Blood samples were collected from 41 patients while they were taking their current medications and from 20 controls.

### miRNA profiling

Whole blood was collected in PAXgene blood RNA tubes (BD Biosciences, Bedford, MA). RNA was isolated using a PAXgene blood miRNA kit (Qiagen, Valencia, CA) following the manufacturer's protocol. RNA concentration was measured using Nanodrop 1000 (NanoDrop Technologies, Wilmington, DE).

Taqman Low Density Array (TLDA) microfluidic cards version A and B (Applied Biosystems, Foster City, CA) were used to profile miRNAs and the protocol recommended by the vendor was followed. We used 50 ng of total RNA in each reaction for cDNA synthesis using a TaqMan microRNA reverse transcription kit and human megaplex RT primers for Pool A and Pool B. Preamplification was done using TaqMan preamplification master mix and human megaplex preamplification primers corresponding to Pool A and Pool B. TLDA cards were assayed on an ABI PRISM 7900 Sequence detector using universal thermal cycling conditions of 50°C for 2 minutes, 95°C for 10 minutes, then 40 cycles of 95°C for 15 seconds and 60°C for 1 minute. The threshold level for background detection in SDS software was manually set to 0.2.

### Data analysis

Quantile normalization was applied to the cycle threshold (CT) values. Samples with CT values 32 and above were treated as undetected as recommended by the vendor. Fold change was calculated from raw CT values using the 2^-ΔΔCT ^method [27]. The mean of the CT values of the 10 miRNAs with the lowest standard deviation was used as the endogenous control in the calculation of ΔCT. Statistical significance of differences in ΔCT values between CRPS patients and controls was calculated by a 2-tailed independent samples *t*-test. The Benjamini-Hochberg false discovery rate correction [28] was applied to the *p*-values. Pairwise Spearman correlation was calculated between various clinical markers and miRNAs. Hierarchical clustering of miRNAs and samples was performed along with the generation of a heatmap of miRNA expression. The samples were clustered into three groups on the basis of their miRNA expression levels and the correlations of other variables against these three groups were calculated.

### Determination of Cytokines/Chemokines and Their Soluble Receptors

The plasma was separated by centrifugation (3000 *g *for 15 minutes at 4°C), split into 250 μL aliquots and stored at -70°C. The Milliplex Map high sensitivity 10 plex human cytokine kit (Millipore, Billerica, MA) was used to determine plasma levels of the following cytokines: interferon-gamma (IFNγ); the interleukins IL-1β, IL-2, IL-4, IL-5, IL-6, IL-7, IL-8, and IL-10; and tumor necrosis factor alpha (TNFα). The Milliplex Map human soluble cytokine receptor panel (Millipore, Billerica, MA) was used to determine the following soluble receptors: soluble glycoprotein 130 (sgp130) (a subunit of the IL-6 receptor complex); the interleukin soluble receptors sIL-1RI, sIL-1RII, sIL-2Rα, sIL-4R and sIL-6R; the TNFα soluble receptors sTNFRI and sTNFRII; and sRAGE, the soluble receptor for advanced glycation end products (AGEs). The plasma levels of the interleukin-1 receptor antagonist (IL1Ra) and the chemokine monocyte chemotactic protein-1 (MCP1) were determined with the Fluorokine MAP Multiplex Human Cytokine Panel A (R&D Systems, Minneapolis, MN). Assay results were determined on a Luminex-200 (Luminex, Austin, TX).

## Results

The average age of controls and patients was 42 ± 12.7 and 45 ± 11.7 years, respectively. The female to male ratio was 14/6 for controls and 27/14 for patients with CRPS. Average body mass index (BMI) for controls and patients was 24.12 and 28.58 respectively indicating normal BMI for control and obesity in our CRPS patient population.

### miRNA alterations in CRPS

**Figure 1:**
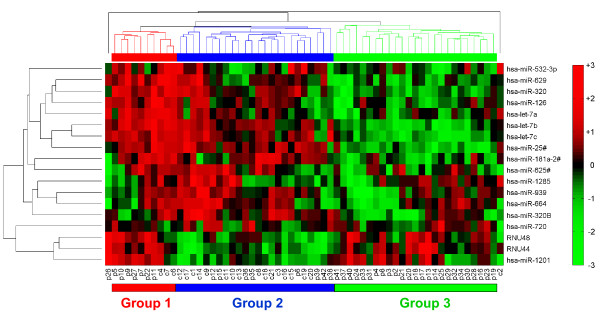
**Differential expression of miRNAs in blood from CRPS patients and control samples**. A clustergram of the samples and miRNAs identifying sample subpopulations with respect to the significant differentially expressed miRNAs. A total of 60% of the patients (Group 3) showed differential regulation for these miRNAs (mostly downregulation), whereas the rest of the patients were more heterogeneous and clustered together with the control samples. P and C represent patients and controls, respectively. Red, high; black, average; green, low.

Two-tailed *t*-tests were used to identify differential expression of miRNAs between patients and control samples. The fold changes and *p *values of significantly altered miRNAs are shown in Table 1. A clustergram of the samples and miRNAs identifying sample subpopulations with respect to the significant differentially expressed miRNAs is shown in Figure 1 as a heat map of the log-transformed fold changes. Heat maps are commonly used for visualization of high-dimensional data in a two-dimensional image with colors representing the intensity values. It is typically used in gene expression analysis to represent the level of expression of many genes across a number of comparable samples. Clustering based on the fold change of significant miRNAs resulted in a clear separation of patient-only population from the rest of the subjects. A total of 60% of the patients (the right half of clustering) showed differential regulation for these miRNAs (mostly down regulation), whereas the rest of the patients were more heterogeneous and were clustered together with the control samples which showed considerable variability.

**Table 1:** Fold changes and *p *values of significantly altered miRNAs

*miRNA*	*Fold change*	*p value*
hsa-miR-939	-4.59358	5.55E-06

hsa-miR-25#	-3.92328	1.09E-06

hsa-let-7c	-2.53502	2.07E-05

hsa-let-7a	-2.45923	0.002308

hsa-let-7b	-2.40344	5.49E-05

hsa-miR-320B	-2.0527	6.91E-06

hsa-miR-126	-2.02362	0.002469

hsa-miR-629.A	-1.71231	0.000653

hsa-miR-664	-1.54881	0.001487

hsa-miR-320	-1.44273	7.28E-05

hsa-miR-1285	-1.41594	0.003077

hsa-miR-625#	-1.33174	0.003542

hsa-miR-532-3p	-1.27226	0.001226

hsa-miR-181a-2#	-1.25927	0.000229

RNU48	1.348125	0.000391

hsa-miR-720	1.476853	0.003243

RNU44	1.854213	0.000904

hsa-miR-1201	2.14584	3.17E-05

Fold changes and *p *values of significantly altered miRNAs (minus sign indicates down-regulation; data sorted based on fold change). The statistical significance was calculated using 2-tailed t-tests on the miRNA expressions in CRPS patients versus control samples.

### Inflammatory markers

**Figure 2:**
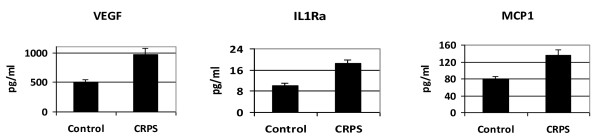
**Elevation of inflammatory markers in CRPS patients**. VEGF, IL1Ra, and MCP1 showed significant differential expression in CRPS patients vs. control samples, with *p *values 0.0002, 0.0004, and 0.0005, respectively. The error bars show the standard error of the means.

Analysis of inflammatory markers including chemokines, cytokines and their soluble receptors in blood from the same individuals showed changes in several markers (Additional file 1). Though there were trends, *t*-tests (Benjamini-Hochberg adjustment of *p *values for multiple comparisons) showed that only VEGF, ILR1a and MCP1 levels were significantly elevated in CRPS patients (Figure 2). VEGF and ILR1a were significantly correlated with the reported pain levels of the patients. VEGF also had a significant correlation with BMI (Additional file 1).

### Analysis of subset of patients with CRPS

Since 60% of the CRPS patients segregated based on miRNA profile (Figure 1 group 3), we analyzed this subgroup of patients further. Additional miRNAs that were identified to be significantly altered are shown in Table 2. For inflammatory and immune related markers, correlations of significance were observed for IL-6, IL-8, IL1Ra, MCP1 and VEGF when considering all CRPS patients versus control samples. Comparing subpopulation group3 against other samples identified additional correlations for IL-4, IL-5, and TNFalpha. However IL-6, IL-8 and MCP1 were not significant in group 3. Thus, stratifying the patient population on the basis of the miRNA profile resulted in changes, predominantly toward the identification of additional indicators that could be clinically relevant in CRPS.

**Table 2:** Significantly altered miRNAs for samples

***Variable***	***p *value**	***Variable***	***p *value**
	
hsa-miR-25#	9.29E-09	hsa-miR-20a#	0.00454
	
hsa-let-7c	4.33E-08	hsa-miR-16-1#	0.005137
	
hsa-let-7b	7.35E-08	hsa-miR-106b	0.00535
	
hsa-miR-320	6.06E-07	hsa-miR-532-3p	0.00535
	
hsa-miR-181a-2#	6.64E-05	hsa-miR-142-5p	0.005419
	
hsa-miR-190b	0.0006	hsa-miR-342-5p	0.005419
	
hsa-miR-221	0.000721	has-miR-155	0.00595
	
hsa-miR-629	0.002053	hsa-miR-130b	0.007069
	
hsa-miR-93	0.002053	hsa-miR-185	0.007069
	
hsa-miR-590-5p	0.002053	hsa-miR-181c	0.007069
	
hsa-miR-29b-2#	0.00306	hsa-miR-20b	0.007154
	
hsa-miR-181a	0.003087	hsa-let-7a	0.009386
	
hsa-miR-144#	0.003087	hsa-miR-422a	0.009471
	
hsa-miR-196b	0.003908	hsa-miR-340	0.009501
	
hsa-miR-132	0.004131	hsa-miR-324-3p	0.009501
	
hsa-miR-652	0.004131	hsa-miR-598	0.009975
	
hsa-let-7g	0.004305	hsa-let-7d	0.009975

Significantly altered miRNAs for samples that clustered in Group 3, based on the miRNA profiling. The statistical significance was calculated using 2-tailed t-tests on the miRNA expression levels in patients that were in Group 3 versus other CRPS patients and control samples.

### Correlation analysis

**Figure 3:**
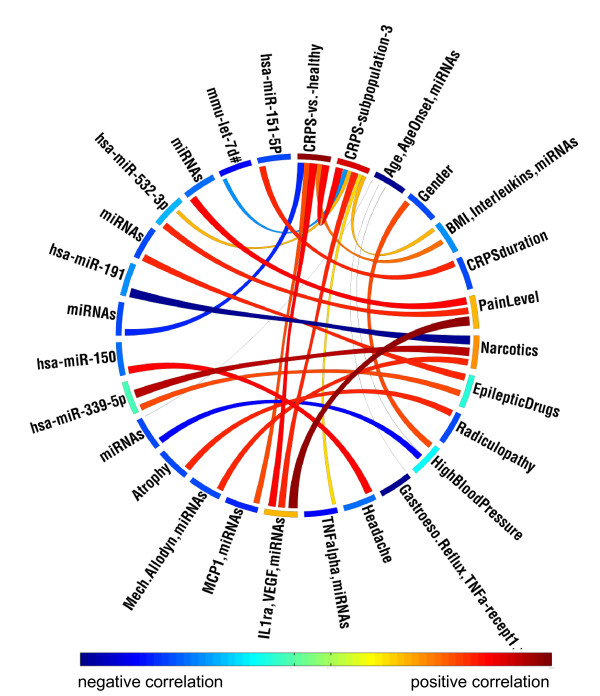
**Circos diagram showing the correlation of selected parameters and miRNAs**. The Circos diagram showing the correlation of selected medical conditions with other clinical parameters and miRNAs. The nodes along the circle are colored by the total strength of correlation of the corresponding variable, such that variables with many strong correlations are shown in red. The links between variables indicate their correlation. Only correlations with adjusted *p *value less than 0.01 are shown. Groups of miRNAs showing similar correlations with other variables are labeled as "miRNAs" for brevity. The links are colored by the Spearman's correlation value, with strong negative correlations shown in darker blue (e.g., narcotics vs. hsa-miR-191) and strong positive correlations shown in darker red (e.g., pain level vs. "IL1ra, VEGF, miRNAs"), as implicated by the color-bar. See the Supplementary table 1 for a complete list of correlations.

We performed additional correlation analyses of all miRNAs detected in whole blood with other parameters including comorbidities. In addition to being in the list of 18 miRNAs identified to be differentially expressed in patients with CRPS, hsa-miR-532-3p was associated with CRPS type, pain level, IL1Ra, and VEGF (Table 3 and Additional file 1). CPRS Type-2 patients from our study had higher hsa-miR-532 and higher VEGF levels compared to CRPS Type-1 patients. We observed a strong correlation between miRNAs and comorbidities such as high blood pressure, cholesterol, thyroid disease, and use of narcotics and antiepileptic medications (Table 3 and Additional file 1). These miRNAs did not overlap with the miRNAs that were modulated in CRPS (Table 1). Though not our primary objective, these results identified miRNA alterations that may be specific to comorbid conditions observed in patients with CRPS. A Circos diagram [29] capturing the significant correlations among all parameters analyzed are shown in Figure 3.

**Table 3:** Correlation studies in CRPS patients for miRNAs, comorbidities and medication.

*Variable 1*	*Variable 2*	*Correlation*	*p value*
**CRPS type**	hsa-miR-532-3p	0.4448	0.00782

**CRPS duration**	hsa-miR-151-5P	0.45992	0.00574

**Pain level**	hsa-miR-296-5p	0.50218	0.00222
	
	hsa-miR-361-3p	0.48489	0.00333
	
	hsa-miR-532-3p	0.46868	0.00478
	
	hsa-miR-30d	0.43451	0.00957

**Inmamov **(inability to initiate and maintain fine movements)	hsa-miR-218	0.46241	0.00546

**High blood pressure**	hsa-miR-92a	-0.5583	0.0005
	
	hsa-miR-484	-0.4422	0.00822

**High cholesterol**	hsa-miR-219-1-3p	-0.4847	0.00334
	
	hsa-miR-204	0.46688	0.00497

**Headache**	hsa-miR-150	0.49815	0.00245

**Thyroid disease**	hsa-miR-616	0.57271	0.00039
	
	hsa-miR-548d-3p	0.48307	0.00391
	
	hsa-miR-27a	0.45319	0.00733
	
	hsa-miR-345	0.45319	0.00733
	
	hsa-miR-324-5p	0.45319	0.00733
	
	hsa-miR-192	0.45319	0.00733
	
	hsa-miR-28-5p	0.44821	0.00808
	
	hsa-miR-502-5p	0.44821	0.00808

**Hyposthesia**	hsa-let-7c	0.45198	0.00677
	
	hsa-miR-500	-0.4379	0.00897

**Narcotics**	hsa-miR-339-5p	0.54176	0.0008
	
	hsa-miR-191	-0.5248	0.00125
	
	hsa-miR-191#	0.49502	0.00264
	
	hsa-miR-486-3p	0.45677	0.00614

**EpilepticDrugs**	hsa-miR-339-3p	0.56671	0.00039
	
	hsa-miR-30c	0.49587	0.00258
	
	hsa-miR-192	0.4917	0.00285
	
	hsa-miR-140-3p	0.48337	0.00344
	
	hsa-miR-152	0.4667	0.00498
	
	hsa-miR-339-5p	0.45003	0.00704
	
	hsa-miR-145#	0.43337	0.00978

**BMI**	hsa-miR-296-5p	0.41312	0.00239
	
	hsa-miR-1290	-0.3842	0.00507
	
	hsa-miR-423-5p	0.38146	0.00544
	
	hsa-miR-25#	0.3774	0.00599
	
	hsa-miR-1270	0.35921	0.00918

All additional correlations observed including inflammatory markers, groups1, 2 and 3 based on miRNA profiling, clinical parameters etc. are shown in Supplementary table 1

## Discussion

We observed differential expression of 18 miRNAs in whole blood from patients with CRPS compared to control samples. Thus multiple miRNAs were significantly different between patients and control subjects compared to three inflammatory and immune related markers. Clustering of 60% of patients with CRPS on the basis of the miRNA profile suggests that clinically relevant stratification of the patient population is possible on the basis of alterations in miRNA expression. miRNAs recognize their target mRNAs using the 2-8 nucleotide sequence at the 5' region of the miRNA called the seed sequence. Target prediction algorithms use different parameters to provide candidate target genes for miRNAs [30]. Our earlier success with TargetScan [23] led us to use TargetScan [31] to perform our initial analysis for miRNAs identified to be differentially expressed in CRPS (data not shown). Bioinformatic prediction of the significantly altered miRNAs showed that these miRNAs can potentially modulate mRNAs of a number of genes relevant in CRPS including inflammatory mediators, ion channels, and G protein-coupled receptors. For example, a bioinformatics-based prediction indicates that hsa-miR-939 can target vascular endothelial growth factor A (VEGF A), inducible nitric oxide synthase 2A, and the alpha subunit of voltage-gated sodium channel type IV and that hsa-miR-25 can target endothelin receptor type B. Since one of the predicted gene targets for hsa-miR-939 is VEGF A, the upregulation of VEGF in the serum of CRPS patients strengthens the prediction. Additional studies including reporter gene assays to validate these predictions and functional consequences of miRNA alterations can provide mechanistic insight into the mode of action of miRNAs in CRPS.

The miRNAs shown in Figure 1 from the CRPS study were compared with miRNAs altered in some of the rodent models for pain investigated. While there was no overlap in the miRNAs identified from studies that focused on a limited number of miRNAs, [19-22,25] profiling from dorsal root ganglion (DRG) from the rat spinal nerve ligation (SNL) model [23] showed that the expression of four miRNAs hsa-miR-126, hsa-let-7a, hsa-let-7b and hsa-let-7c (multiple copies of let-7 family of miRNAs are present in the genome; isoforms are distinguished by a letter placed after let-7 to indicate a slightly different sequence) was significantly altered in both CRPS blood and rat DRG after SNL. Profiling of 369 miRNAs was performed in rat DRG 4 weeks post SNL [23], whereas the current study profiled 758 miRNAs. Thus, not all miRNAs identified in the present CRPS study have been profiled in rat DRG. This finding is an indication of the potential translational value that can be achieved from miRNA profiling.

Expression of small nucleolar RNAs (snoRNAs) RNU44 and RNU48 was found to be altered in CRPS patients. RNU44 and RNU48 have been widely used for miRNA data normalization, but a recent study reported that normalizing miRNA expression data to these recommended snoRNAs introduced bias in associations between miRNA and pathology or outcome [32]. Their function is not well understood, but recent reports suggest that the noncoding growth arrest-specific transcript 5 gene (*GAS5*), which encodes multiple snoRNAs, is significantly downregulated in breast cancer [33]. *GAS5 *is necessary and sufficient for the arrest of T-cell growth and for the inhibitory effects of rapamycin and its analogues. It has been suggested that these effects may be mediated through the snoRNAs. These observations have important clinical implications because these compounds are used in immunosuppression and in cancer therapy [34].

Though the pathophysiology of CRPS is not completely understood, it is known that neurogenic inflammation plays a significant role [35]. Studies suggest that trauma-induced release of inflammatory cytokines facilitates neurogenic inflammation [35,36]. Mast cells, neutrophils and macrophages are recruited to the injured area and because of the compromised blood-nerve barrier they invade the nerve [37]. These cells release proinflammatory cytokines that have been implicated in the generation of neuropathic pain either by direct sensitization of nociceptors or indirectly by stimulating the release of agents that act on neurons and glia [38,39]. Thus both neuroinflammation and neuroimmune activation act in concert in persistent pain states [40]. The balance between pro- and anti-inflammatory cytokines may ultimately determine the chronic pain state [41]. An increase in the level of proinflammatory cytokine IL-2 and a decrease in the level of anti-inflammatory cytokines IL-4 and IL-10 were reported in CRPS [42,43]. However, systemic inflammatory cytokine responses have been inconsistent between the different study populations and settings. Trends toward an increase in proinflammatory cytokines (IL-6, IL-8, TNF alpha) and a decrease of the anti-inflammatory cytokine IL-10 in CRPS subjects compared with the controls have been observed but none of the changes reached statistical significance [44]. It has not yet been established clinically that measurement of plasma cytokines is helpful in the diagnosis or follow-up of CRPS patients. However, in a recent study using a large patient population, clustering patterns showed that plasma cytokine levels should not be evaluated in isolation and that their effect may differ depending on the plasma level of their soluble receptors and receptor antagonists [45]. When all CRPS patients were analyzed only VEGF, MCP1 (CCL2) and IL1Ra were significantly regulated whereas grouping patients based on miRNA profiling (Group 3) resulted in additional markers that were significantly altered including TNFalpha, IL-4 and IL-5. miRNAs associated with comorbidities and medications (Table 3) were different from those associated with CRPS.

We observed hsa-mir-150 to be correlated with headache and several other miRNA correlations with comorbidities such as high blood pressure, thyroid disorder, use of medications including narcotics and antiepileptic drugs. These results indicate the broader utility of performing miRNA profiling and could provide additional molecular insights into disease biology occurring as comorbidities or use of specific medications.

Our studies indicate that miRNA profiling can serve as a novel approach for patient stratification. Stratification based on the miRNA profile resulted in identification of additional markers that were not significant when all CRPS patients were analyzed as a single group. Stratification of patients can be clinically relevant in CRPS and additional patterns could emerge with increase in sample size. The potential for identifying several miRNAs as signatures rather than relying on one specific biomarker will increase the chances of successful treatment in the heterogeneous CRPS patient population. Identifying informative benchmarks will be an exceptionally valuable tool for assisting physicians in choosing treatment options and for stratifying patients in clinical trials. By performing similar miRNA profiling in animal models to cross validate the human data, we can gain further insight into mechanistic aspects of CRPS. miRNAs or the genes they modulate can be direct targets for future therapeutic interventions. Bridging preclinical and clinical results could provide new insights into the molecular mechanisms underlying chronic pain.

The functional relevance of the presence of stable miRNAs in blood is an area of active investigation. A recent study demonstrated a novel mechanism of intercellular communication involving the transport and delivery of miRNAs [46]. Intercellular communication was thought to be limited to cell-to-cell adhesion conduits (gap junctions) or secreted signals such as hormones and neurotransmitters; however, it has been shown that miRNAs are transported in plasma and delivered to recipient cells by high-density lipoproteins resulting in modulation of target mRNAs [46]. Delivery of miRNAs to recipient cells with functional gene regulatory consequences via blood opens up novel avenues for target intervention. Identification and functional validation of mRNAs modulated by miRNAs altered in the blood of patients with CRPS can thus demonstrate the potential for miRNA to regulate the expression of pain-relevant genes. These studies can lead to a greater understanding of the molecular mechanisms involved in chronic pain and potentially determine if any of the miRNAs or the genes they modulate can be direct targets for future therapeutic interventions.

## Conclusions

The ability to cluster 60% of the patients with CRPS suggests that miRNA profiling can serve as a novel, clinically relevant approach for patient stratification. Grouping patients based on miRNA profiling resulted in the identification of additional markers that were not significant when the whole CRPS population was considered. This could be due to the etiology as well as the multitude of pathologies and symptoms associated with CRPS. Correlational studies linking miRNA changes with inflammatory markers and clinical parameters provided a broad overview of the interplay of multiple factors critical in CRPS. Additional studies validating the role of the miRNAs in gene regulation can help to obtain insights into molecular mechanisms underlying CRPS.

## List of abbreviations

3'-UTR: 3' untranslated region of messenger RNAs; AGEs: advanced glycation end products; BMI: body mass index; CRPS: complex regional pain syndrome; CT: cycle threshold; DRG: dorsal root ganglion; hsa-mir: Homo sapiens-microRNA; IL: interleukins; IL1Ra: interleukin1 receptor antagonist; MCP1: monocyte chemotactic protein-1; NRS: numerical rating scale; sgp130: soluble glycoprotein 130; sIL: soluble interleukin receptors; SNL: spinal nerve ligation; sTNFR: soluble tumor necrosis factor receptor; TLDA: Taqman low density array; TNFα: tumor necrosis factor alpha; VEGF: vascular endothelial growth factor.

## Competing interests

The authors declare that they have no competing interests. A provisional patent has been filed relating to the content of the manuscript.

## Authors' contributions

IO carried out the miRNA profiling and analyzed the data; GA participated in study design, study supervision and coordination, data acquisition of inflammatory markers, analysis and interpretation of data, and drafted the manuscript; RQ performed bioinformatics and statistical analysis, revised the manuscript; AS performed and coordinated bioinformatics, statistical analysis, data interpretation, and drafted the manuscript; AG performed statistical analysis, interpretation of data and revised the manuscript, JB participated in study design, data interpretation and revising the manuscript; RS performed all the clinical exams, study supervision and coordination in the clinic, revised the manuscript; SA conceived the study concept, performed study supervision, coordination, data analysis, interpretation of data and wrote the manuscript. All authors read and approved the final manuscript.

## Supplementary Material

Additional file 1 **Correlation studies for miRNAs, comorbidities and medication**. Significant correlations observed including 1) miRNA and clinical parameters, 2) miRNA and inflammatory markers, 3) miRNA and groups, 4) inflammatory and immune related markers, and 5) clinical parameters are shown in sheets 1, 2, 3, 4 and 5 respectively. Groups in sheet 3 includes CRPS vs. healthy as well as CRPS subpopulation 1, CRPS subpopulation 2 and CRPS subpopulation 3 representing group 1, 2 and 3 based on differential expression of miRNAs. Click here for file
